# Multifunctional glucose biosensors from Fe_3_O_4_ nanoparticles modified chitosan/graphene nanocomposites

**DOI:** 10.1038/srep11129

**Published:** 2015-06-08

**Authors:** Wenjing Zhang, Xiaojian Li, Ruitao Zou, Huizi Wu, Haiyan Shi, Shanshan Yu, Yong Liu

**Affiliations:** 1 Lab of Nanoscale Biosensing and Bioimaging, Institute of Advanced Materials for Nano-Bio Applications, School of Ophthalmology & Optometry, Wenzhou Medical University, 270 Xueyuan Xi Road, Wenzhou, Zhejiang 325027, China; 2 Advanced Cytometry Labs, ARC Center of Excellence for Nanoscale BioPhotonics, Macquarie University, Sydney, NSW 2109, Australia

## Abstract

Novel water-dispersible and biocompatible chitosan-functionalized graphene (CG) has been prepared by a one-step ball milling of carboxylic chitosan and graphite. Presence of nitrogen (from chitosan) at the surface of graphene enables the CG to be an outstanding catalyst for the electrochemical biosensors. The resulting CG shows lower I_D_/I_G_ ratio in the Raman spectrum than other nitrogen-containing graphene prepared using different techniques. Magnetic Fe_3_O_4_ nanoparticles (MNP) are further introduced into the as-synthesized CG for multifunctional applications beyond biosensors such as magnetic resonance imaging (MRI). Carboxyl groups from CG is used to directly immobilize glucose oxidase (GO_x_) via covalent linkage while incorporation of MNP further facilitated enzyme loading and other unique properties. The resulting biosensor exhibits a good glucose detection response with a detection limit of 16 μM, a sensitivity of 5.658 mA/cm^2^/M, and a linear detection range up to 26 mM glucose. Formation of the multifunctional MNP/CG nanocomposites provides additional advantages for applications in more clinical areas such as *in vivo* biosensors and MRI agents.

Magnetic nanoparticles (MNP), due to their biocompatibility, strong superparamagnetic property, and low biotoxicity, have attracted promising interests for applications in diverse biomedical areas such as drug delivery, hyperthermia treatment, cell separation and biosensors^1^,^2^,^3^,^4^. Particularly biosensors for detection of H_2_O_2_ has been assembled from Fe_3_O_4_ nanoparticles modified carbon electrodes^5^,^6^. A high-performance glucose biosensor has been prepared from chitosan/Fe_3_O_4_ nanocomposites^7^. Even numerous reports suggested potential applications of Fe_3_O_4_ nanoparticles as catalysts for electrochemical biosensors, their catalytic activities are limited by their finite electrochemical activity. Graphene and its derivatives have commonly been considered as the excellent substrates for biosensor architectures since their unique surface area, electronic conductivity and stability^8^,^9^,^10^, though electrochemical catalytic activity of graphene are still required for improvement. Fe_3_O_4_ nanoparticles (NP) were thus incorporated with graphene for biosensor applications. For example, a H_2_O_2_ biosensor was prepared from Fe_3_O_4_ NP deposited on the reduced graphene oxide sheets (Fe_3_O_4_/RGO). The detection sensitivity was found to be 0.0468 μA μM^−1^ linear up to 1 mM^11^. The biosensor performance, however, is poor due to limited catalytic activity of the Fe_3_O_4_/RGO nanocomposites. Chitosan is the second most abundant natural polymer next to cellulose^12^, which has been considered as the most promising substrate for enzyme immobilization due to its unique biocompatibility and multiple functional groups^13^. Combination of biocompatible chitosan and conductive graphene is thus considered as a good strategy for design of high-performance biosensors. A NO biosensor was established from hemoglobin (Hb) immobilized chitosan and graphene with presence of surfactant hexadecyltrimethylammonium bromide (CTAB). A sensitivity of 0.615 μA μM^−1^ was obtained^14^. A cholesterol biosensor was prepared by immobilization of cholesterol oxidase (ChOx) onto chitosan modified graphene via situ reduction of chitosan and microwave synthesized graphene oxide^15^. A linear detection of cholesterol in the range of 0.005–1 mM was identified. A high-performance H_2_O_2_ biosensor was also synthesized from microperoxidase-11 (MP-11) immobilized chitosan/graphene nanocomposite with a sensitivity of 0.77 μA mM^−1^ 16. Fe_3_O_4_ was further introduced into chitosan/graphene based biosensors for multifunctional applications. The biosensing performance, however, was decreased significantly and the linear range was only up to 1.67 mM^17^. Challenges for introduction of MNP while remaining good performance of chitosan/graphene based biosensors have attracted increasing attention. Improvement of catalytic activity of chitosan/graphene composites via structural modification has been considered as a promising resolution for these issues.

Recently, different types of nitrogen doped graphene (N-G) with highly electrochemical activity have been reported by various techniques such as chemical vapor deposition (CVD)^18^,^19^, chemical post-treatment of graphene oxide^20^,^21^, plasma modifications^22^, and microwave enhancement^23^. Presence of atom-acceptor nitrogen in the carbon conjugated matrix has found to influence the charge distribution on the surrounding carbons, providing superb active sites for electrochemical catalysis^24^. It has been reported that N-G could be used as the catalysts for high-performance biosensors^25^. Besides introduction of nitrogen atoms into carbon matrix, nitrogen-containing groups such as nitrobenzene has been surface doped with graphene and exhibited highly catalytic activity for oxygen reduction^26^. To date, few publications have reported biocompatibility of N-G based catalysts for biosensors which is essential for development novel *in vivo* biosensors. It is thus interesting to design nitrogen-containing biomaterials (e.g. chitosan) doped graphene for biosensor applications. Incorporation of nitrogen (from chitosan) may provide highly catalytic activity for sensing performance while presence of chitosan can improve biocompatibility of resulting electrodes, providing suitable environment for enzyme immobilization.

In this work, we presented a facile but efficient way to synthesize nitrogen-containing chitosan doped graphene (C-G) for electrochemical biosensors using a one-step ball milling technique^27^,^28^. In addition, we incorporated Fe_3_O_4_ nanoparticles with CG for multifunctional applications. Combination of MNP and CG not only combined magnetic properties with catalytic activity but also provided additional advantages for the hybrid materials such as larger active surface areas and enhanced electron transport with formation of 3D hybrids from nanoparticle modified nanosheets which are useful for fabrication of electrochemical sensing devices^29^,^30^,^31^. In this article, we have immobilized glucose oxidase into the Fe_3_O_4_/CG hybrids via covalent linkage to build up high-performance electrochemical biosensors for detection of glucose. The resulting hybrids can be further used for multifunctional applications beyond biosensors such as MRI imaging.

## Results

**Figure 1:**
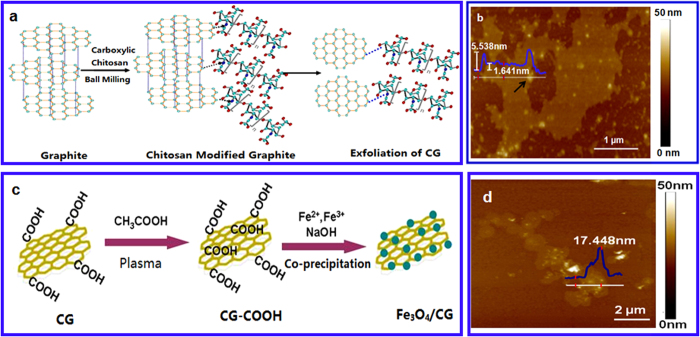
Formation of the Fe_3_O_4_/CG nanocomposites. **a** Schematic synthesis of the CG. **b** A typical AFM image of the CG nanosheets. Arrow indicates presence of chitosan. **c** Schematic preparation of the Fe_3_O_4_/CG nanocomposites. **d** A typical AFM image of the Fe_3_O_4_/CG nanocomposites.

Formation of the CG via ball milling is schematically shown in Fig. 1(a). Chitosan will edge-functionalize graphite sheets at the initial step. The increasing chitosan chains and amounts along with the milling shear forces will lead to the chain breaking between graphite sheets, facilitating exfoliation of graphene nanosheets. The as-prepared CG was further modified using acetic acid plasma treatment to introduce plenty of active carboxyl-functional groups for Fe_3_O_4_ nanoparticle loading (Fig. 1(c)). Figure 1(b,d) show AFM images of the CG and Fe_3_O_4_/CG nanomaterials. The as-synthesized graphene nanosheets are found to be around 1.641 nm (Fig. 1(b)), suggesting single-/few layer of chitosan functionalized graphene nanosheets can be prepared using the ball milling technique as we reported previously^28^. Presence of chitosan the on CG is shown as the arrow indicated in Fig. 1b. The total thickness of CG is around 5.538 nm. Excellent water-dispersibility of the as-synthesized CG is shown in Figure S1 (Supplementary Information, SI). Good dispersion of CG is remained well even after storing in air over 15 days. Well distribution of Fe_3_O_4_ nanoparticles on the CG nanosheets is observed in Fig. 1(d). The thickness of the Fe_3_O_4_/CG nanocomposites is about 17.448 nm, indicating that the average diameter of the Fe_3_O_4_ nanoparticles is about 12 nm. Morphology of the resulting Fe_3_O_4_/CG hybrids was further measured by TEM. As shown in Figure S2 (SI), well distributed nanoparticles are homogeneously and uniformly decorated on the surface of the CG nanosheet which showing a typical flake-like shape. The average diameter of Fe_3_O_4_ nanoparticles on the nanosheets is found to be about 12 nm, well consistent with AFM results.

**Figure 2:**
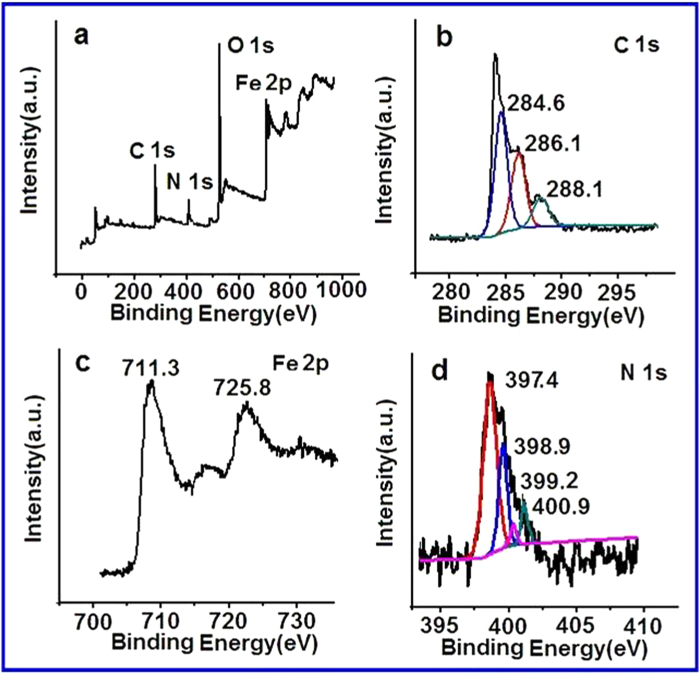
XPS spectra of the Fe_3_O_4_/CG nanocomposites. **a** XPS survey spectrum of the Fe_3_O_4_/CG nanocomposites. **b** The high-resolution C1s spectrum. **c** The high-resolution Fe2p spectrum. **d** The high-resolution N1s spectrum.

XPS results of the resulting Fe_3_O_4_/CG hybrids are shown in Fig. 2. The XPS survey spectrum of the resulting Fe_3_O_4_/CG nanocomposites indicates three elements besides O e.g. N content at 400 eV, the C signal at 284 eV, Fe at 710 eV and 725 eV (Fig. 2a), confirming the successful combination of CG nanosheets and Fe_3_O_4_ nanoparticles. The nitrogen content in the resulting nanocomposites is found to be 5.16%. The high-resolution C1s spectrum (Fig. 2c) shows three dominated peaks associated with sp^2^ hybridized C atoms (284.6 eV), the C-NH_2_ (286.1 eV) and sp^3^ C atoms bonded with N and O (288.1 eV) respectively^23^. The Fe2p emission spectrum (Fig. 2d) shows two peaks at 711.3 eV and 725.8 eV which are related to Fe2p_3/2_ and Fe2p_1/2_ respectively, confirming the formation of Fe_3_O_4_. The high-resolution N 1 s spectrum is fitted by four peaks (Fig. 2d). The predominant peak at 397.4 eV is arisen from the nitrogen in chitosan which is confirmed by the high-resolution N 1 s spectrum of the pristine chitosan as shown in Figure S3 (SI). Presence of both pyridinic nitrogen (398.9 eV) and pyrrolic nitrogen (399.2 eV) is revealed within the CG structure, providing active sites for electrochemical catalysis^32^,^33^. The peak at 400.9 eV is associated with quaternary nitrogen.

**Figure 3:**
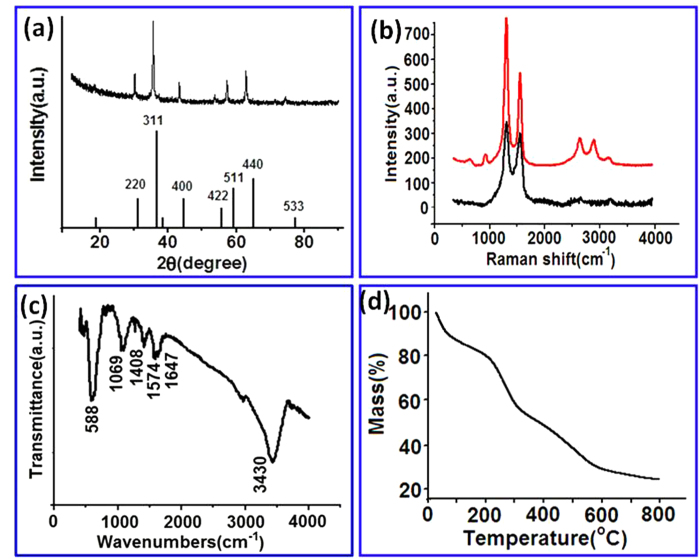
Physicochemical characterization of the Fe_3_O_4_/CG nanocomposites. **a** XRD patterns of the Fe_3_O_4_/CG. **b** Raman spectra of the CG and the Fe_3_O_4_/CG. **c** The FTIR spectrum of the Fe_3_O_4_/CG. **d** The TGA curve of the Fe_3_O_4_/CG.

Figure 3(a) shows the XRD patterns of the resulting Fe_3_O_4_/CG nanocomposites. The intense diffraction peaks at 30.1°, 36.8°, 43.2°, 54.1°, 58.9° and 63.7°, are indexed to (220), (311), (400), (422), (511) and (440) respectively. The peak positions and relative intensities match well with inverse spinel structure of magnetite [JCPDS: 19-0629], confirming the presence of Fe_3_O_4_. No obvious diffraction peak attributed to graphite is observed, indicating that the graphene sheets in the Fe_3_O_4_/CG nanocomposites are disordered^34^. Raman spectra of the CG and Fe_3_O_4_/CG nanocomposites both exhibit characteristic D band, G band and 2D band for graphene (Fig. 3(b)). The D band at 1350 cm^−1^ arises from the vibrations of sp3 carbon atoms while the G band at 1595 cm^−1^ represents the E2g mode of sp^2^ carbon atoms in a 2D hexagonal lattice^35^. The relative intensity ratio of the D band to G band (I_D_/I_G_ ratio) is proportional to the content of defect sites in graphite carbon^36^. The I_D_/I_G_ of the as-prepared CG is 1.15, much lower than the reported nitrogen-doped graphene prepared using other methods including chemical treatment^37^, microwave^23^, and plasma^25^, suggesting the edge-functionalized ball milling technique can be used to prepare graphene nanosheets with fewer defects which is well consistent with previous work regarding nitrogen-edge functionalized graphene nanoplatelets prepared by dry ball milling graphite with N_2_^38^. The I_D_/I_G_ ratio has increased to 1.48 when Fe_3_O_4_ nanoparticles are introduced to the CG nanosheets, suggesting more defects introduced and good interactions between the nanoparticles and nanosheets. Figure 3(c) shows the FTIR spectrum of the Fe_3_O_4_/CG nanocomposites. A strong band at 3430 cm^−1^ is attributable to stretching vibration of N-H bonds, which is further confirmed by the peak at 1574 cm^−1^ arisen from bending vibration of N-H. The peak obtained at 1647 cm^−1^ is associated with C = O stretching and the peak at 1408 cm^−1^ is probably related to scissoring and bending of C-H. The peak at 1069 cm^−1^ is due to the stretching vibration of C-N bond. The band at 588 cm^−1^ is related to Fe–O functional groups evidenced as the characteristic peak for Fe_3_O_4_^39^. The FTIR spectrum confirms that the Fe_3_O_4_ nanoparticles have been successfully deposited on the CG nanosheets. TGA was used to evaluate the mass ratio of Fe_3_O_4_ in the Fe_3_O_4_/CG hybrid. As shown in Fig. 3(d), the weight loss (10%) step between 50–150 °C might be due to the loss of residual water and adsorbed organics in the sample. The weight loss (62%) from 150 to 600 °C is associated with the loss of CG nanosheets. The content of residue Fe_3_O_4_ is found to be about 28%. So the mass ratio of Fe_3_O_4_ to CG is about 1:2.

**Figure 4:**
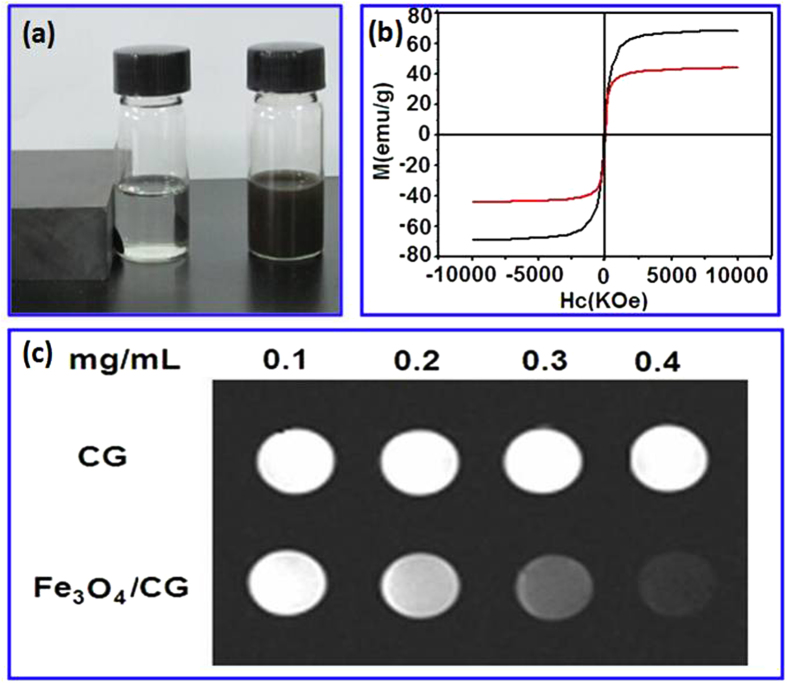
Magnetic properties of the Fe_3_O_4_/CG nanocomposites. **a** Digital photos of the Fe_3_O_4_/CG nanocomposite suspension with and without an exterior magnetic field. **b** magnetic hysteresis curve of the Fe_3_O_4_ and Fe_3_O_4_/CG nanomaterials. **c** T_2_ weighted MRI images of the CG and Fe_3_O_4_/CG nanomaterials.

The magnetic controlled movement of the Fe_3_O_4_/CG hybrid is illustrated in Fig. 4(a). Strong attraction of the nanocomposites towards the external magnet is evident, suggesting readily separation of the nanocomposites out of the dispersion. Figure 4(b) shows magnetic hysteresis loops of the resulting nanomaterials measured using superconductive quantum interference device (SQUID) over the range of −10 < H < 10 kOe at room temperature. Superparamagnetic properties of the Fe_3_O_4_/CG hybrids (42 emu/g) are obtained, comparable to that of the pristine Fe_3_O_4_ (68 emu/g). Magnetization results show that the as-synthesized Fe_3_O_4_/CG hybrid remained excellent magnetic properties of the pristine Fe_3_O_4_, suggesting their possible applications such as the MRI imaging. Figure 4(c) shows the T_2_-weighted MR images of the CG compared to the Fe_3_O_4_/CG hybrid at 3.0 T on a Trio Tim Imager. It is found that the CG sample shows less dark contrast. Deposition of Fe_3_O_4_ nanoparticles to CG, however, causes decrease in brightness of T_2_-weighted MR image. This may be attributed to that magnetic nanoparticles change the magnetic relaxation properties of nearby water protons, resulting in reduction of the T2 relaxation time.

**Figure 5:**
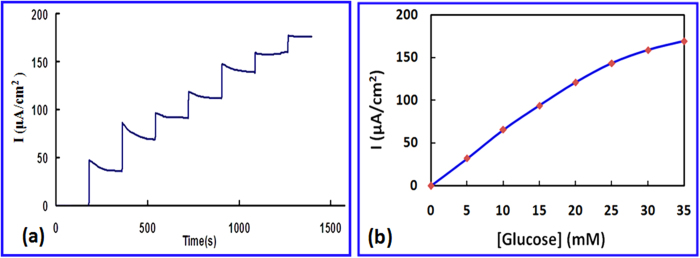
Biosensor performance. **a** Amperometric responses of the Fe_3_O_4_/CG -GOx electrode to successive additions of 5 mM of glucose at 0.5 V *vs* Ag/AgCl in 0.1 M PBS (pH = 7.4). **b** the calibration curve obtained for glucose detection.

GO_x_ was further introduced onto the Fe_3_O_4_/CG hybrid for the purpose of glucose detection. As shown in Figure S4 (SI), there is no obvious redox peaks observed in the cyclic voltammogram (CV) of the Fe_3_O_4_/CG without addition of glucose. A significant couple of redox peaks due to the redox reaction between glucose and GO_x_ are identified when 10 mM glucose is introduced in the electrolyte (0.1 M PBS (pH = 7.4) ), confirming the excellent detection responsibility of the resulting enzyme electrode to glucose. For the purpose of comparison, different individual components including the pristine Fe_3_O_4_ NP, the as-synthesized CG, ball milling treated graphite without incorporation of chitosan, and the Fe_3_O_4_/CG are used for GOx immobilization and tested using cyclic voltammetry in glucose containing PBS solution (Figure S5, SI). No redox behaviour is observed at the pristine Fe_3_O_4_ NP and ball milling treated graphite electrode. A much lower redox response is obtained at the CG electrode when compared to the resulting Fe_3_O_4_/CG electrode. Results suggest that introduction of chitosan provides both nitrogen as the active catalytic sites and suitable microenvironment for enzyme immobilization while incorporation of Fe_3_O_4_ further enhance enzyme loading. Current responses at the Fe_3_O_4_/CG-GO_x_ on the successive addition of 5 mM glucose at a constant potential of +0.5 V (vs. Ag/AgCl) are shown in Fig. 5(a). It is found that the oxidation currents increases significantly with addition of glucose while no current changes are observed at the pristine Fe_3_O_4_/CG electrode, indicating that the oxidation currents are associated to the oxidation of hydrogen peroxide arising from the enzyme reaction rather than the direct oxidation of glucose. Figure 5(b) shows the calibrated steady current responses with respect to accumulative glucose concentrations added. The sensitivity of the Fe_3_O_4_/CG hybrid based glucose biosensor is found to be 5.658 μA/cm^2^/mM (equals to 5.658 mA/cm^2^/M) determined by the slope of the calibration curve, three times higher than that of the Fe_3_O_4_/CG nanocomposite without plasma treatment based biosensor (Figure S6, SI), confirming that plasma treatment is necessary for enzyme loading. The current responses are linear up to 26 mM, much higher than the blood glucose concentration (15 mM) required for clinical detection. The detection limit of the as-synthesized biosensor is found to be 16 μM. The reproducibility of the resulting biosensor is obtained from 8 parallel enzyme electrodes prepared at identified conditions. A relative standard deviation (RSD) of 5.59% is obtained, suggesting excellent reproducibility. The long-term stability of the resulting biosensors was evaluated by storing the enzyme electrode at 4 °C for 30 days. 24.3% decrease in sensitivity is obtained during the biosensor testing, suggesting well long-term stability of the biosensor. The decreased sensitivity may be attributed to deactivation of the GOx during long-term storage.

## Discussion

Heteroatom doped graphene particularly N-G has attracted extreme intensive attention during recent years due to its superb properties esp. electrochemical catalytic activities. Though numerous techniques including CVD, chemical treatment, plasma and microwave have been developed for preparation of N-G, recently reported edge-functionalized ball milling method has been considered as a novel and essential way to prepare graphene derivatives due to its environmentally friendly, facile and highly efficient process. Presence of atom-acceptor nitrogen surrounding the π-π conjugated carbon facilitates charge transferring from surrounding carbon to nitrogen, resulting in active sites for electrochemical catalytic reactions such as glucose oxidation by GOx. On the other hand, development of graphene based biosensors enhances biosensor performance (e. g. sensitivity, detection limit, linear detection range) significantly, which opens up great possibilities for clinic *in vivo* diagnosis and therapy using nanostructured biosensors/biochips. Besides sensing behaviors, biocompatibility of the enzyme electrode is also essential for enzyme immobilization and future *in vivo* biosensor establishment. Combination of well biocompatible and biodegradable biomaterials such as chitosan with highly active N-G is thus seen as an efficient strategy for future biosensor design.

We have prepared excellent active N-G catalysts via a fast and efficient way by ball milling of chitosan and graphite. Process of ball milling grinds chitosan into small active molecules which edge-functionalize graphite sheets and expand layered spaces of graphite layers during the initial steps of ball milling. The covalent bonds between graphite layers are weaken by the increasing amount of chitosan molecules introduced which facilitate exfoliation of graphene nanosheets with the synergistic effects of ball milling shear force. Presence of nitrogen (from chitosan) around carbon conjugated matrix provides not only active sites for biosensing but also excellent biocompatibility for both enzyme immobilization and *in vivo* applications.

In addition, complicated *in vivo* environment and limitation of traditional diagnosis promote demands for design of multi-modal diagnosis techniques such as multifunctional biosensors. In this work, we have incorporated superparamagnetic Fe_3_O_4_ NP into the as-synthesized CG for preparation of high-performance biosensors with additional MRI applications. Incorporation of Fe_3_O_4_ NP into the CG further increases surface areas of the nanosheests which is beneficial for enhanced enzyme loading and electron transport between the enzyme and the electrode.

## Conclusion

Our preliminary work presents a facile but efficient way to prepare novel water-dispersible nitrogen containing biomaterials chitosan modified graphene using a one-step ball milling technique. The as-synthesized CG nanosheets exhibit single to few layered thickness and highly catalytic activity for biosensors. Magnetic nanoparticles Fe_3_O_4_ is further introduced to the as-synthesized CG for the purpose of enhanced enzyme immobilization, electrochemical activity and additional magnetic properties. The resulting Fe_3_O_4_/CG hybrid based biosensor has been assembled. A highly sensitivity (5.658 mA/cm^2^/M) with a low detection limit (16 μM) and broad linear detection range up to 26 mM is achieved. The resulting biosensor also shows good reproducibility and long-term stability with additional advantages of applications in different areas such as MRI imaging, opens up possibilities for fabrication of novel multifunctional nanobiosensors for future clinic multimodal diagnosis and therapy.

## Methods

### CG nanosheest

CG was prepared by mixing graphite and carboxylic chitosan (1:20 w/w) in a ball milling capsule. The mixture was vigorously shaken at a speed of 500 rpm/min for 12 h prior to be removed out by deionized (DI) water for centrifugation at 8000 rpm for 10 min. The upper solid was collected after centrifugation and dialyzed in DI water overnight for removal of any impurities.

### Fe_3_O_4_/CG hybrid nanomaterials

Formation of the Fe_3_O_4_/CG hybrid was schematically shown in Fig. 1(c). A plasma treatment under acetic acid was carried out on the CG to introduce more active carboxyl groups for deposition of nanoparticles. Fe_3_O_4_/CG nanocomposites were then fabricated using the co-precipitation technique^15^. Typically, the as-synthesized CG was well dispersed in DI water at concentration of 0.5 mg/mL and remained at 80 °C with magnetic stirring. FeCl_3_.6 H_2_O (30 mg) and FeCl_2_.4 H_2_O (380 mg) was then added in the dispersion above while the mixture was remained at 80 °C with stirring overnight under N_2_. 3 mol/L NaOH was then dropwisely introduced, followed by further magnetic stirring for 3 h at 80 °C. The resulting Fe_3_O_4_/CG nanocomposites were consequently obtained after washing ethanol and water three times, and drying in oven at 60 °C for 12 h.

### Fabrication of the enzyme electrode

Pt sputter coated ITO glass (100 mA/cm^2^, 30 s) was used as the working electrode. The Pt coated ITO was subsequently immersed in the 0.5 mg/mL^−1^ Fe_3_O_4_/CG dispersion for 3 h and dried at the room temperature overnight. The resulting electrode was then immersed in a PBS solution (pH = 7.4) containing 34 mg/mL EDC and 17 mg/mL NHS over 2 h at room temperature to active carboxyl groups at the CG. GOx was subsequently immobilized into the Fe_3_O_4_/CG nanocomposites by immersing the Fe_3_O_4_/CG into 5 mg/mL GOx/0.1 M PBS solution (pH = 7) at 4 °C for 2 h.

### Electrochemical measurements

Electrochemical measurements were conducted using a CHI 760D electrochemical workstation with conventional three-electrode setup at room temperature. A platinum wire and a Ag/AgCl (saturated KCl) electrode was used as the counter and reference electrode respectively. Amperometric response were measured in 0.1 M PBS (pH = 7.4) at a constant potential of +0.5 V where hydrogen peroxide produced from the oxidation of glucose is oxidized. Oxidation current response with successive additions of 5 mM glucose was recorded.

## Additional Information

**How to cite this article**: Zhang, W. *et al.* Multifunctional glucose biosensors from Fe_3_O_4_ nanoparticles modified chitosan/graphene nanocomposites. *Sci. Rep.* **5**, 11129; doi: 10.1038/srep11129 (2015).

## Supplementary Material

Supplementary Information
