# Supplementary material for: Multifunctional glucose biosensors from Fe3O4 nanoparticles modified chitosan/graphene nanocomposites
Source: Sci Rep. 2015 Jun 8;5:11129. doi: 10.1038/srep11129 (PMC4459104; doi:10.1038/srep11129)
Supplement: Supplementary Information [file srep11129-s1.doc]

**Supplementary Information**

**Multifunctional glucose biosensors from Fe_3_O_4_ nanoparticles modified chitosan/graphene nanocomposites**

Wenjing Zhang,^1^ Xiaojian Li,^1^ Ruitao Zou,^1^ Huizi Wu,^1^ Haiyan Shi,^1^ Shanshan Yu,^1^ and Yong Liu^1,2^,*

*^1^Lab of Nanoscale Biosensing and Bioimaging,Institute of Advanced Materials for Nano-Bio Applications, School of Ophthalmology & Optometry, Wenzhou Medical University, 270 Xueyuan Xi Road, Wenzhou, Zhejiang 325027, China*

*^2^Advanced Cytometry Labs, ARC Center of Excellence for Nanoscale BioPhotonics, Macquarie University, Sydney, NSW 2109, Australia*

*Email: yongliu1980@hotmail.com

**Materials**

Ferric chloride (FeCl_3_.6H_2_O), ferrous chloride (FeCl_2_.4H_2_O), sodium hydroxide (NaOH), carboxylic chitosan and graphite were purchased from Sinopharm Chemical Reagent Co., Ltd. Glucose and glucose oxidase (GOx) was purchased from Sigma-Aldrich. 0.1 M phosphate buffer solutions (PBS) consisted of Na_2_HPO_4_ and NaH_2_PO_4_, were employed as supporting electrolyte. The desired solution pH was adjusted by different amount of 0.1M Na_2_HPO_4_ and NaH_2_PO_4_ solutions. Well deionized water was used in all experiments.

**Characterization**

A Digital Instruments Mutimode 8 (Bruker, German) atomic force microscopy (AFM) in tapping mode was used to identify thickness and morphology of the resulting nanomaterials. Transmission electron microscopy (TEM) images were obtained by a JEM 2010 instrument. X-ray diffraction (XRD) patterns were measured by a Scintag XDS2000 goniometer equipped with a graphite monochromator. The data were recorded at room temperature over the 2θ range from 5 to 90 ^o^ with a scanning rate of 2.0 ^o^ min^-1^. X ray photoemission spectroscopy (XPS) studies were performed by ESCALAB 250 (ThermoFisher SCIENTIFIC, England) using the monochromatic Al Kα radiation. All spectra were calibrated to the binding energy of the C 1speak at 284.6 eV. The Raman spectra were obtained by a Renishaw 2000, which detector use an air-cooling charge-coupled device, and equipped with an Ar^+^ ion laser at the excitation line of 514.5 nm. Fourier transform infrared (FTIR) spectra were determined using a PE Spectrum One spectrometer with the scan region from 400 to 4000 cm^-1^. Thermogravimetric analysis (TGA) was carried out using a TA Q500 (Waters, America) with a heating rate of 10 ^o^C min^-1^. A Quantum Design vibrating sample magnetometer (mmvFTB) was used to evaluate vibrating sample magnetization (VSM).

**Results**

**
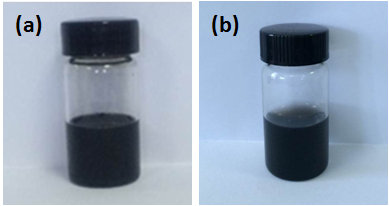
**

Figure S1. Digital photos of water dispersion of (a) the newly prepared CG, and (b) the as-synthesized CG after storing in air over 15 days.

**
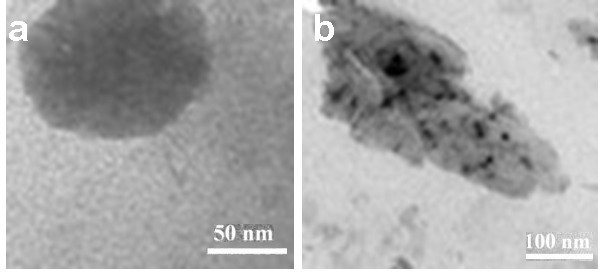
**

Figure S2. TEM images of (a) the CG and (b) the Fe_3_O_4_/CG nanocomposites.


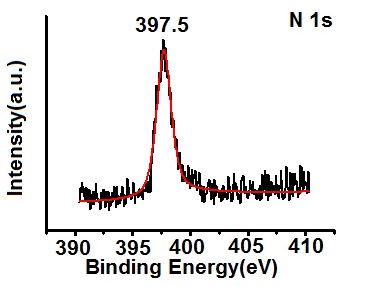


Figure S3. The high-resolution XPS N1s spectrum of the pristine chitosan.


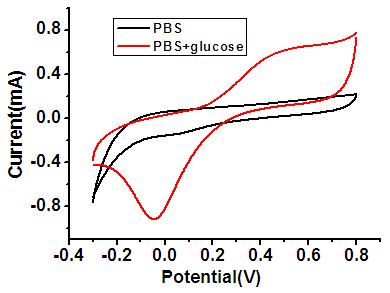


Figure S4. Cyclic voltammograms of the Fe_3_O_4_/CG-GOx electrode in 0.1M PBS solution (pH=7.4) without and with of 10 mM glucose. Potential scan rate: 100 mV/s.


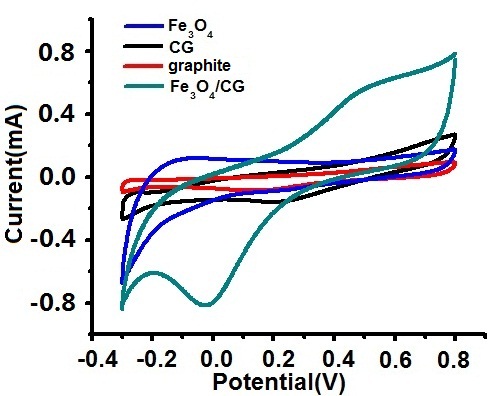


Figure S5. Cyclic voltammograms of various electrodes in 10 mM glucose/0.1M PBS solution (pH=7.4). (a) Fe_3_O_4_ nanoparticles, (b) CG, (c) graphite without chitosan after ball milling, and (d) Fe_3_O_4_/CG nanocomposites. Potential scan rate: 100 mV/s.


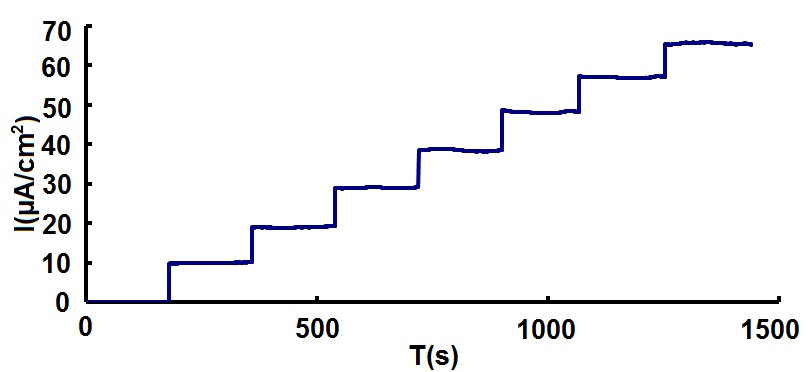


Figure S6. Amperometric responses of the Fe3O4/CG -GOx electrode without plasma treatment to successive additions of 5 mM of glucose at 0.5V vs Ag/AgCl in 0.1 M PBS (pH=7.4). Sensitivity=1.905 mA/cm^2^/M.
